# Morphological and Phylogenetic Analysis of *Eustrongylides* sp. and *Gnathostoma spinigerum* Parasitizing the Asian Swamp Eel *Monopterus* *albus* in China

**DOI:** 10.3390/pathogens10060711

**Published:** 2021-06-07

**Authors:** Sixin Zhang, Guangping Huang, Liang Li, Xianyong Liu, Xiaoli Tang, Xun Suo

**Affiliations:** 1 National Animal Protozoa Laboratory, College of Veterinary Medicine, China Agricultural University, Beijing 100193, China; zhangsixin@cau.edu.cn (S.Z.); liuxianyong@cau.edu.cn (X.L.); tangxiaoli@fuwaihospital.org (X.T.); 2 Key Laboratory of Animal Ecology and Conservation Biology, Institute of Zoology, Chinese Academy of Sciences, Beijing 100101, China; huanggp@ioz.ac.cn; 3 Key Laboratory of Animal Physiology, Biochemistry and Molecular Biology of Hebei Province, College of Life Science, Hebei Normal University, Shijiazhuang 050016, China; liliang745@hebtu.edu.cn

**Keywords:** *Eustrongylides*, *Gnathostoma spinigerum*, ITS, COI, *Monopterus albus*, China

## Abstract

Nematode infections transmitted to humans by the consumption of wild or cultured eels are increasingly being reported. In the present study, 120 Asian swamp eel, *Monopterus albus* (Zuiew), individuals collected from China were examined for parasite infections, and 78 larval nematodes were isolated. Morphological and molecular characteristics, including sequence and phylogenetic analysis of the internal transcribed spacer (ITS) and cytochrome c oxidase subunit I (COI) gene regions, were employed to identify these nematodes at the lowest taxonomic level possible. Asian swamp eel was infected with two zoonotic parasite taxa: *Gnathostoma spinigerum* advanced third-stage larvae, with 6.67% prevalence and mean intensity = 1.25, and *Eustrongylides* sp. fourth-stage larvae, with 26.67% prevalence and mean intensity = 2.13. These findings evidence the need to enhance public hygiene and food safety awareness toward eel consumption

## 1. Introduction

The Asian swamp eel *Monopterus albus* (Zuiew) (Synbranchiformes: Synbranchidae) is widely distributed in Asia, including India, China, Japan, and Malaysia. In Southeast Asia and China, where live individuals are sold in many urban fish markets and widely consumed as common food [1,2]. In addition to being one of the most commercially important fish species, the Asian swamp eel serves as an intermediate or paratenic host for various nematode parasites, including *Gnathostoma*, acanthocephalans, trematodes, cestodes, trypanosome, metacercariae of digenea [3,4,5,6,7,8,9]. In recent years, many eels were transported alive from Asia to Australia, Africa, and Central or South America, posing a high zoonotic threat to global health [10,11,12].

In Asian countries, cultured and wild swamp eels have a high prevalence of infection by the parasitic nematodes *Gnathostoma* spp. and *Eustrongylides* spp., both known sources of zoonotic diseases, widely distributed, and having a complex life-cycle that involves invertebrates (such as cephalopods and oligochaetes) and vertebrates (e.g., fishes, mammals, and birds). Among the 12 species of *Gnathostoma* spp., five species have been reported to infect humans. *G. spinigerum* is commonly distributed in China, India, Japan, and Southeast Asia; *G. hispidum* is mainly found in Asia, Australia, and Europe; *G. doloresi* existed in Southeast Asia; *G. nipponicum* is found in Japan and Korea; and *G. binucleatum* is distributed in Mexico and some South American countries. There are three species in the genus of *Eustrongylides* spp. including *E. tubifex, E. ignotus,* and *E. excisus*, which are found in several species of fish and oligochaetes and the intestine of water birds. [13,14,15,16,17,18,19,20,21,22,23,24]. Digestive disorders and intestinal perforation can occur in humans due to the accidental ingestion of third- or fourth-stage *Eustrongylides* spp. larvae via consumption of raw or undercooked parasitized fish [13,14,25,26,27]. Advanced third-stage *Gnathostoma* spp. larvae cause migratory swellings under the skin and can enter other tissues such as liver and eye tissues, resulting in vision reduction or blindness, and sometimes cause nerve pain, paralysis, coma, or even death [8,21,22]. In China, the first human case of gnathostomiasis was reported in 1925; since then, more than 58 cases have been sporadically reported [18]. Although human eustrongylidosis has not been reported in China, at least six cases were found in Japan, Thailand, Sudan, and the United States, indicating a potential infection risk in China [13,14,26].

The clinical signs and symptoms of gnathostomiasis and eustrongylidosis vary widely and can be easily confused with those of other parasitic infections (such as sparganosis and cysticercosis), resulting in misdiagnoses and treatment delay [23]. Therefore, the accurate identification of the nematodes causing these infections is critical for their diagnosis and treatment. Previous studies indicated that, in addition to morphological identification, molecular markers such as the ITS1, ITS2, and COI, can assist in the epidemiological investigation of parasite species [12,28,29,30,31,32]. Furthermore, evaluating the prevalence of nematode infections among Asian swamp eels will provide data for the effective control of parasitic diseases. Thus, in the present study, the nematode genera infecting the Asian swamp eel in China were identified based on morphological and molecular characteristics, and their prevalence was determined, aiming to generate public hygiene awareness related to eel consumption.

## 2. Results

### 2.1. Morphological Characterization

#### 2.1.1. *Gnathostoma spinigerum*

The most useful characteristic for identifying *Gnathostoma* sp. larvae is the cephalic bulb because this character is easily observed. However, body length, the number of rows with hooklets in the cephalic bulb, and the number of rows with spines on the body surface can also be used to identify *Gnathostoma* sp. larvae.

**Figure 1:**
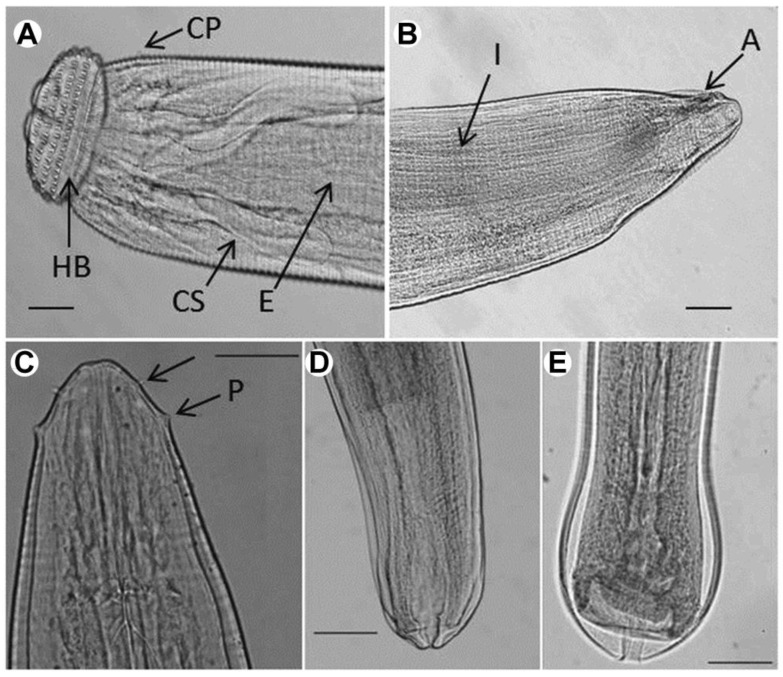
Photomicrographs of *Eustrongylides* sp. and *Gnathostoma spinigerum* larvae (lateral view). (**A**) Anterior part of *G*. *spinigerum*. Four rows of hooklets extruded from the surface of the cephalic bulb; (**B**) posterior end of *G*. *spinigerum*; A: anus; CP: cervical papilla; CS: cervical sac; E: esophagus; HB; head bulb; P: papillae. (**C**) Anterior end of *Eustrongylides* sp., showing two rows of papillae; (**D**) posterior end of *Eustrongylides* female; (**E**) posterior end of *Eustrongylides* male. *Scale-bar* = 50 µm.

The *Gnathostoma* specimens examined in the present study were 3.632 ± 0.594 mm (3.215–4.054 mm) in length, 0.395 ± 0.019 mm (0.382–0.409 mm) in maximum width, and the body was covered with more than 200 transverse rows of single apex spines (4.647 ± 1.024 μm in length). Four rows of hooklets extruded from the surface of the cephalic bulb. The cephalic bulb was 0.108 ± 0.018 mm (0.095–0.120 mm) long and 0.209 ± 0.019 mm (0.195–0.223 mm) wide. A pair of lateral pseudolabia was observed. The muscular esophagus was almost cylindrical, 1.120 ± 0.028 mm (1.102–1.141 mm) in length, and connected to the intestine. Two pairs of cervical sacs, 0.451 ± 0.028 mm (0.423–0.508 mm) long, were found around the esophagus. One pair of lateral cervical papilla was located at 0.350 ± 0.055 mm (0.306–0.420 mm) from the anterior extremity to. The anus, 0.062 ± 0.014 mm (0.055–0.067 mm) in length, was located at the posterior end. The tail was very short, measuring only 0.184 ± 0.037 mm (0.182–0.188 mm) (Figure 1A,B and Table 1).

Overall, these characteristics were consistent with the original description of *G. spinigerum* advanced third-stage larva (AL3; Owen, 1836), including larvae body length, number of rows with hooklets, and transverse single apex spines [2,33].

#### 2.1.2. *Eustrongylides* sp.

Most parasitic specimens encysted in the mesentery of swamp eels presented a cylindrical body with delicate transverse striations on the surface (Figure 1C). The anterior one-third of the larval body was pink-red, while the remaining portion was bright red. Two circles of cephalic papillae surrounded the mouth, each bearing six papillae: two lateral, two sub-ventral, and two sub-dorsal. The cephalic papillae distributed in the inner circle had narrow bases and sharp apices, whereas the outer ones had wide bases and rounded apices. So the parasite might be *Eustrongylides* sp. fourth-stage larvae [3].

The *Eustrongylides* specimens examined in the present study had the following characteristics: Females (*n* = 6) were 57.675 ± 3.172 mm (55.220–61.350 mm) in length and 0.502 ± 0.048 mm (0.466–0.566 mm) in maximum width. The inner-circle papillae were 0.022 ± 0.018 mm (0.016–0.040) away from the oral opening, and the outer-circle papillae were 0.066 ± 0.012 mm (0.032–0.079 mm) from the anterior end. The nerve ring was at 0.325 ± 0.059 mm (0.279–0.382 mm) from the anterior end and the muscular esophagus at 14.311 ± 1.152 mm (11.253–18.090 mm). The tail was 0.250 ± 0.062 mm (0.210–0.292 mm) in length. The vulvar primordium was 6.150 ± 0.680 mm (4.950–6.690 mm) from the posterior end and was well developed (Figure 1C,D and Table 2). Males (*n* = 6) were 55.450 ± 3.136 mm (53.150–61.250 mm) in length and 0.419 ± 0.032 mm (0.398–0.430 mm) in maximum width. The inner-circle papillae were at 0.022 ± 0.015 mm (0.016–0.032 mm) from the oral opening, and the outer-circle papillae at 0.062 ± 0.017 mm (0.041–0.0643) from the anterior extremity. The nerve ring was at 0.313 ± 0.035 mm (0.287–0.334 mm) from the anterior end and the muscular esophagus at 14.091 ± 0.523 mm (13.252–14.920 mm). The tail was 0.236 ± 0.074 mm (0.216–0.260 mm) long (Figure 1C,E and Table 2). Overall, these measurements were consistent with the genus-specific parameters previously described for *Eustrongylides* sp. [30].

### 2.2. ITS and COI Amplification and Sequencing

The ITS2 (including 5.8S complete sequence and 28S partial sequence) and COI sequences of *Gnathostoma* sp. obtained were 568 and 726 bp in length, respectively, and both displayed 99% nucleotide similarity with *G. spinigerum* sequences deposited in GenBank (Accessions KF648531–KF648553 and AB037132).

Six different sequences were obtained for the ITS of the *Eustrongylides* sp. larvae examined here, ranging from 877 to 900 bp in length and differing by 1–2%. Their alignment with *Eustrongylides* sp. XF-2009 (Accessions GQ215499–GQ215579) retrieved from GenBank revealed more than 95% similarity. The COI sequence obtained was 419 bp long and displayed over 94% nucleotide similarity with *Eustrongylides* sp. XF-2009 (Accessions GQ215580–GQ215653).

### 2.3. Phylogenetic Analysis

**Figure 2:**
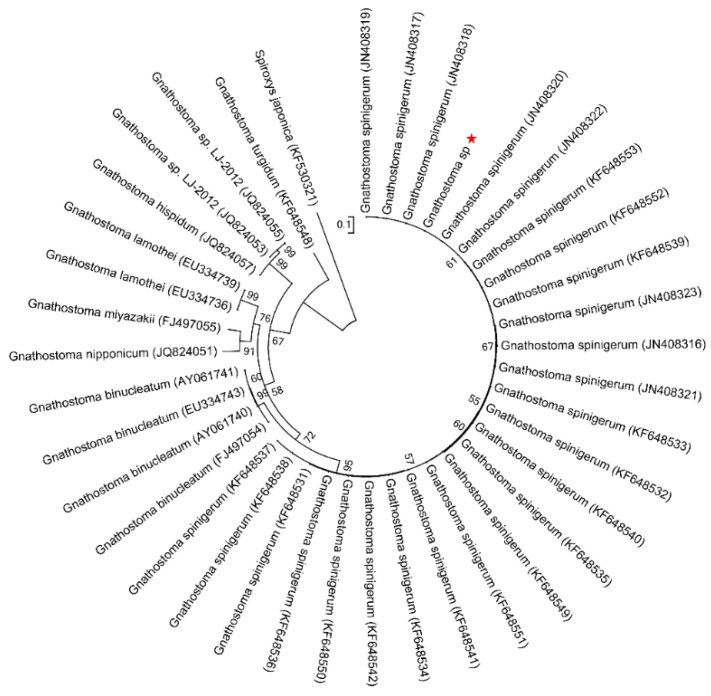
ML tree estimated for the ITS2 region of *Gnathostoma* nematodes using *Spiroxys japonica* (Accession KF530321) as outgroup. Nodal values refer to 1000 bootstrap replicates support.

**Figure 3:**
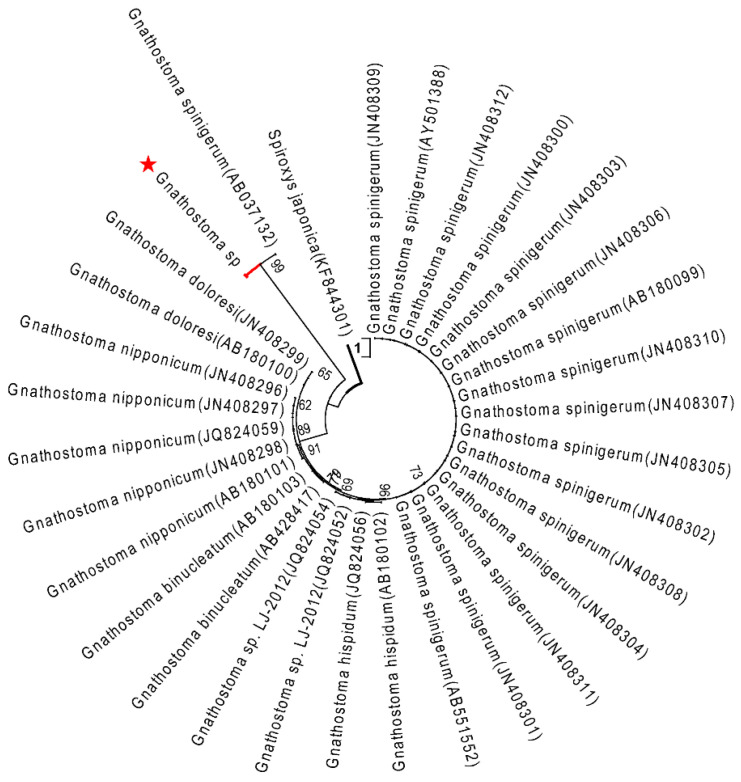
ML tree estimated for the COI region of *Gnathostoma* nematodes using *Spiroxys japonica* (KF844301) as outgroup. Nodal values refer to 1000 bootstrap replicates support.

The topology of the maximum likelihood (ML) phylogenetic tree of *Gnathostoma* sp. based on an extensive *Gnathostoma* sp. ITS2 sequences dataset and using *Spiroxys japonica* (Accession KF530321) as outgroup was highly similar to previously published Gnathostomatiidae phylogenies (Figure 2). The *Gnathostoma* sp. sequences obtained here clustered with *G. spinigerum* (Accessions KF648531–KF648553 and JN408316–JN408323). In the ML tree constructed for COI sequences and using *Spiroxys japonica* (Accession KF530325) as an outgroup (Figure 3), two clades were obtained for *G. spinigerum*.

**Figure 4:**
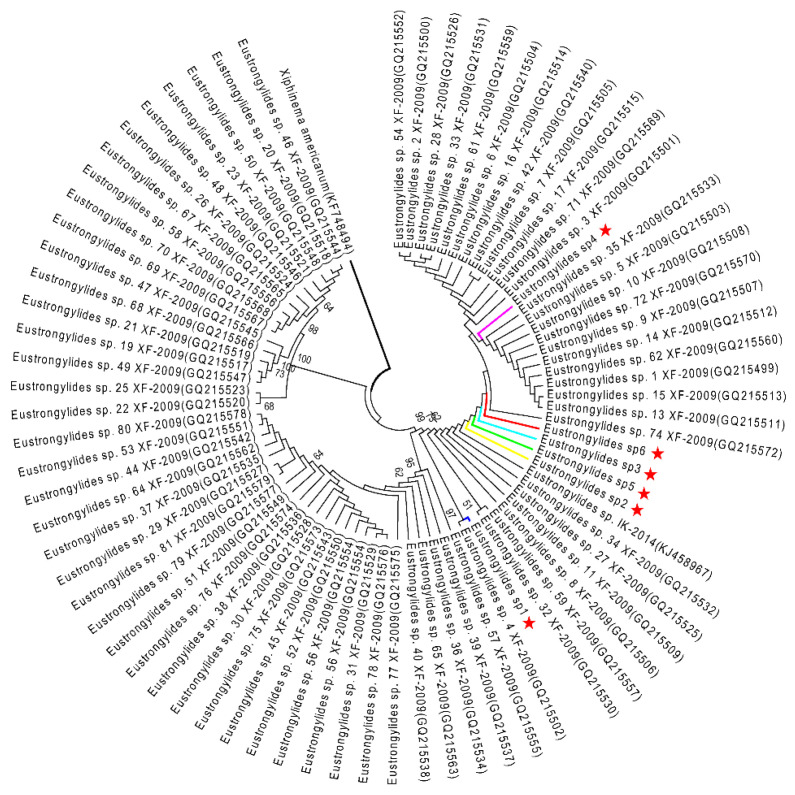
ML tree estimated for the ITS region of *Eustrongylides* nematodes using *Xiphinema americanum* (KF748494) as outgroup. Nodal values refer to 1000 bootstrap replicates support.

**Figure 5:**
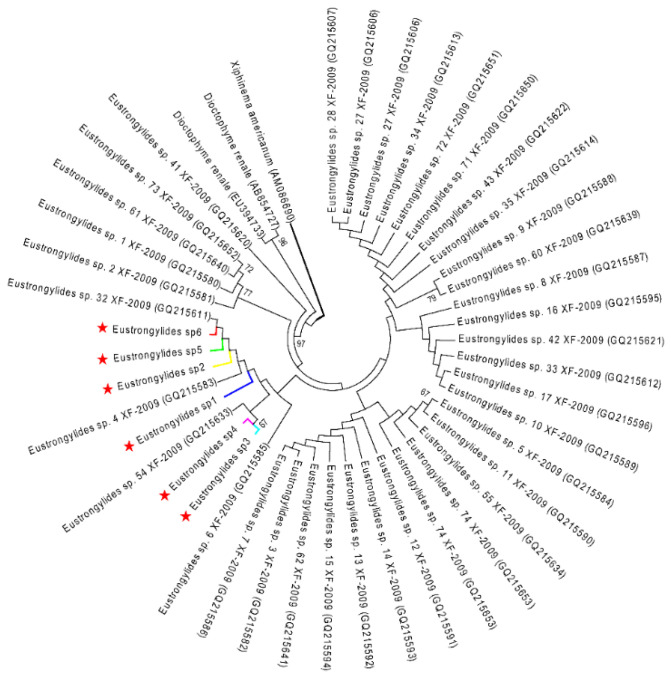
ML tree estimated for the COI region of *Eustrongylides* nematodes using *Xiphinema americanum* (AM086690) as outgroup. Nodal values refer to 1000 bootstrap replicates support.

The ML analysis of the *Eustrongylides* sp. ITS and COI datasets yielded two very similar trees, each displaying three major clades (Figure 4 and Figure 5). In both trees, the *Eustrongylides* sp. sequences obtained here are grouped with *Eustrongylides* sp. XF-2009 (Accessions GQ215499–GQ215579, 100% bootstrap support in the ITS tree; Accessions GQ215580–GQ215653, 100% bootstrap support in the COI tree).

The phylogenetic analysis of *Gnathostoma* and *Eustrongylides* specimens revealed distinct clusters within *G. spinigerum* and *Eustrongylides* sp. XF-2009, further confirming the morphological and molecular analyses results.

### 2.4. Ecology of Eustrongylides sp. and Gnathostoma Spinigerum in the Asian Swamp Eel

Among the 78 larval nematodes collected, 68 were identified as *Eustrongylides* sp. and 10 as *Gnathostoma spinigerum*. The 68 fourth-stage *Eustrongylides* sp. larvae were recovered from 32 swamp eels: 38 were found in the mesenteries, 18 were found in the visceral cavity, and 12 were found in the midgut. The prevalence and mean intensity of *Eustrongylides* sp. were 26.67% (32 infected eels/120 examined eels) and 2.13 (68 parasites/32 hosts), respectively. Eight swamp eels had 10 in total grossly visible white nodules on their liver or muscle, each containing at least one *G. spinigerum* AL3. The prevalence and mean intensity of *G. spinigerum* were 6.67% (8 infected eels/120 examined eels) and 1.25 (10 parasites/8 hosts), respectively. Notably, six eels (5%, 6/120) were co-infected with these two parasites.

## 3. Discussion

Few epidemiological data are available for Asian swamp eel nematode infections in China [19,34]. In the present study, 78 larval nematodes were collected from 120 Asian swamp eels captured in China and examined immediately after death. Based on morphological and molecular characteristics, two zoonotic nematode taxa were identified: *G. spinigerum* advanced third-stage larvae, with 6.67% prevalence and mean intensity = 1.25, and *Eustrongylides* sp. fourth-stage larvae, with 26.67% prevalence and mean intensity = 2.13.

Accurate species identification should rely on molecular data accompanied by strong morphological evidence acquired from adult nematodes. Although Measures [3] and Xiong [30] recorded and examined adult *Eustrongylides* sp. in fish-eating birds, larvae belonging to this genus are difficult to diagnose due to their similarity with related genera, resulting in taxonomic confusion. For example, the larvae of *Eustrongylides* sp. and its sister genus *Dioctophyme* sp. share some characteristics, such as infection site, body size, and cephalic features [35]. Previous studies demonstrated that molecular genetic markers (such as ITS or COI) are useful tools for larvae identification [27,36,37,38]. However, due to the absence of *Eustrongylides* adult sequences in GenBank, a specific identification could not be assigned. In the present study, both morphological and molecular data supported the identification of the *Gnathostoma* sp. advanced third-stage larva as *G. spinigerum*. The clear segregation of *G. spinigerum* species in the ML tree might result from the nucleotide differences among the COI gene sequences.

A high prevalence (over 40%) of *Eustrongylides* sp. in Asian swamp eel has been reported for Asian countries [10,32]. Previous studies reported a wide variation in the prevalence of *G. spinigerum* AL3: approximately 19.6% in Vietnam [39,40] and 27.6% in the United States [2]. The slightly lower prevalence values found in the present study might be related to the length of the eels examined here (35–45 cm) [41]; meanwhile, the environmental factors, such as distribution of swamp eel, season, and so on, might also influence the prevalence values [39,41,42].

In conclusion, the occurrence of two zoonotic parasites, *G. spinigerum* and *Eustrongylides* sp., within the Asian swamp eel in Sichuan and Hubei provinces in China, contributes to enhancing public hygiene and food safety awareness, as this is a widely consumed fish species. So enough time is needed for cooking to ensure that the larvae in food are killed, and the infection is blocked. The results found in the present study also provide important epidemiological data on nematode infection in commercial fish species in China, while our research is insufficient in the time span of parasites epidemiology of Asian swamp eel.

## 4. Materials and Methods

### 4.1. Parasites Collection

One hundred and twenty live Asian swamp eels, ranging from 35 to 45 cm in total length, were purchased from Jingshen aquatic product markets (39°09′ N, 116°03′ E) in Beijing, China, from May to August 2014. These eels had been captured in Neijiang (29°11′ N, 104°15′ E, Sichuan Province, China) and Xiantao (30°04′ N, 112°55′ E, Hubei Province, China). Eels were sacrificed by anesthetization with tricaine methanesulfonate (MS-222, Sigma-Aldrich Corporation, St. Louis, MO, USA), and thin sections of muscle tissue were dissected, mounted in glass slides, and observed under light microscopy. Additionally, muscle tissue samples were incubated in a digestive solution (1% pepsin solution in HCl, pH 2), at 37 °C for 4–5 h, under continuous stir. The suspension was centrifuged at 3000× *g* for 5 min, and the resulting precipitate was rinsed and resuspended with phosphate buffer (Solarbio, China) before examination under the stereomicroscope (SteREO Discovery.V12, ZEISS, Germany). The liver, kidney, and gastrointestinal tract were removed, separated, and examined for parasites, along with the visceral cavity. Recovered larvae (*n* = 34) were fixed and stored in 70% ethanol with 0.5% glycerin for further study.

### 4.2. Morphological Observation

To visualize the important structures for nematodes’ morphological identification, individuals were cleared in lactophenol as previously described [43,44] and then examined under the microscope. Body length, maximum body width, length from nerve ring to anterior end, esophagus length, and lip size were determined. Measurements are provided in millimeters unless otherwise stated and presented as mean ± SD, followed by the range (in parentheses). Photomicrographs were obtained using a digital optical microscope (Olympus, Tokyo, Japan).

### 4.3. DNA Extraction and Molecular Identification

Genomic DNA was extracted from the middle part of individual worms using a Column Genomic DNA Isolation Kit according to the instructions of the manufacturer (TIANGEN, Beijing, China). The primers (Table 3) for the ITS2 and COI regions were designed. The PCR mix (total volume of 50 μL) contained 50 ng template DNA, 1 μL (50 pmol/μL) of each genus-specific primer, and 25 μL 2 × Taq Mix (GenStar, Beijing, China). The reaction profile included an initial denaturation step at 95 °C for 5 min, 35 cycles at 95 °C for 30 s, 55 °C for 30 s, and 72 °C for 30 s, and a final extension step at 72 °C for 10 min, the PCR product (PCR mix without DNA template) was used as a negative control. After confirming the amplification of the desired DNA fragments through electrophoresis, PCR amplicons were purified reference to the procedures from Easypure Quick Gel Extraction kit (TransGen Biotech, Beijing, China), cloned into pEASY-T vectors (TransGen Biotech, Beijing, China), and confirmed by Sanger sequencing. The consensus DNA sequences obtained were compared to those deposited in the GenBank database using the basic local alignment search tool (https://blast.ncbi.nlm.nih.gov/Blast.cgi, accessed on 27 April 2021).

### 4.4. Phylogenetic Analysis

To determine the phylogenetic relationships between the nematode taxa identified in the present study and their closely related species (based on sequences retrieved from GenBank), ITS and COI maximum likelihood trees were constructed in MEGA (Molecular Evolutionary Genetics Analysis) version 5.1 (CEMI, Tempe, AZ, USA) [46].

*Xiphinema americanum* (AM086690, KF748494) was used to root the phylogenetic trees of *Eustrongylides* sp. [34], while *Spiroxys japonica* (KF530325, KF530321) was used as an outgroup in *Gnathostoma* sp. phylogenetic trees [47,48]. Statistical support for taxa grouping was estimated using bootstrap analysis (1000 replicates).

## Figures and Tables

**Table 1:** Morphometrics (in mm) of the advanced third-stage *Gnathostoma spinigerum* larvae detected in Asian swamp eel.

Characteristic *	AL3 (*n* = 6)
Body length	3.632 ± 0.594
Maximum width	0.395 ± 0.019
Length of cephalic bulb	0.108 ± 0.018
Width of cephalic bulb	0.209 ± 0.019
Esophagus	1.120 ± 0.028
Cervical papillae to anterior end	0.350 ± 0.055
Length of cervical sacs	0.451 ± 0.028
Anus to posterior end	0.062 ± 0.014
Tail	0.037

* All measurements are shown as mean ± standard deviation (SD).

**Table 2:** Morphometrics (in mm) of the fourth-stage *Eustrongylides* sp. larvae detected in Asian swamp eel.

Characteristic *	Males (*n* = 6)	Females (*n* = 6)
Body length	55.450 ± 3.136	57.675 ± 3.172
Max body width	0.419 ± 0.032	0.502 ± 0.048
Internal circle of papillae to anterior end	0.022 ± 0.015	0.022 ± 0.018
External circle of papillae to anterior end	0.062 ± 0.017	0.066 ± 0.012
Nerve ring to anterior end	0.313 ± 0.035	0.325 ± 0.059
Esophagus	14.091 ± 1.523	14.311 ± 1.152
Esophagus/body length (%)	25.408 ± 1.445	25.816 ± 1.488
Tail	0.236 ± 0.074	0.250 ± 0.062
Vulvar primordium to posterior end	–	6.150 ± 0.680

* All measurements are shown as mean ± SD.

**Table 3:** Primers used to obtain ITS and COI amplicons from the *Eustrongylides* and *Gnathostoma* specimens collected in the present study.

Species	Gene	Primers (5–3′)		Annealing Temperature	Target Size (bp)
*Eustrongylides* spp.*	ITS	F R F R	TGGATGATTCGGTGAGGT	55 ℃	900
			AACCGCTTAGTAATATGCT		
	COI		ACNACRTARTANGTRTCRTG	55 ℃	419
			TGRTTYTTYGGNCAYCC		
*Gnathostoma* spp.†‡	ITS	F R F R	TGTGTCGATGAAGAACGCAG	55 ℃	568
			TTCTATGCTTAAATTCAGGGG		
	COI		TTTTGGGCATCCTGAGGTTTAT	55 ℃	726
			AAAGAAAGAACATAATGAAAA		

* The primers used for ITS (18S partial sequence, 5.8S subunit complete sequence, and 28S partial sequence) and COI amplifications were obtained from Xiong et al. (2013) [34]. † The primers used for ITS2 (5.8S subunit complete sequence and 28S partial sequences) amplification were obtained from Cole et al. (2014) [2]. ‡ The primers used for COI amplification were obtained from Hashimoto et al. (1997) [45].

## Data Availability

All the raw data is available and provided upon request.

## References

[1] Froese R., Pauly D. FishBase. https://www.fishbase.se/summary/Monopterus-albus.html.

[2] Cole R.A., Choudhury A., Nico L.G., Griffin K.M. (2014). *Gnathostoma spinigerum* in live Asian swamp eels (*Monopterus* spp.) from food markets and wild populations, United States. Emerg. Infect. Dis..

[3] Measures L.N. (1988). Epizootiology, Pathology, and Description of *Eustrongylides-Tubifex* (Nematoda, Dioctophymatoidea) in Fish. Can. J. Zool..

[4] Chen Q.Q., Lin X.M. (1991). A survey of epidemiology of *Gnathostoma hispidum* and experimental studies of its larvae in animals. Southeast Asian J. Trop. Med. Public Health.

[5] Coyner D.F., Spalding M.G., Forrester D.J. (2003). Epizootiology of *Eustrongylides ignotus* in Florida: Transmission and development of larvae in intermediate hosts. J. Parasitol..

[6] Alvarez-Guerrero C., Muñoz-Guzmán M.A., Buendía-Jiménez J.A., Alba-Hurtado F. (2011). *Gnathostoma binucleatum*: Pathological and parasitological aspects in experimentally infected dogs. Exp. Parasitol..

[7] Bertoni-Ruiz F., Argumedo M.R.L.Y., Garcia-Prieto L., Osorio-Sarabia D., Leon-Regagnon V. (2011). Systematics of the genus *Gnathostoma* (Nematoda: Gnathostomatidae) in the Americas. Rev. Mex. Biodivers..

[8] Defendi G.L. Gnathostomiasis. http://emedicine.medscape.com/article/998278-overview.

[9] Wai A.P., Sripan P., Boonmars T., Sriraj P., Aukkanimart R., Songsri J., Boueroy P., Boonjaraspinyo S., Khueangchaingkhwang S., Laummaunwai P. (2019). Seasonal variation of parasitic infections in Asian swamp eels (*Monopterus albus*) from local markets in Yangon, Myanmar. Vet. Integr. Sci..

[10] Sohn W.M., Kho W.G., Lee S.H. (1993). Larval *Gnathostoma nipponicum* found in the imported Chinese loaches. Korean J. Parasitol..

[11] Haugen P., Hemmingsen W., MacKenzie K. (2008). The distribution of *Eustrongylides* sp (Nematoda: Dioctophymatoidea) in brown trout *Salmo trutta* L. in the River Otra in southern Norway. Bull. Eur. Assoc. Fish Pathol..

[12] Hernández-Gómez R.E., Martínez-Salazar E.A., López-Jiménez S., León-Règagnon V. (2010). Molecular identification of the advanced third-stage larvae (ADV L(3)) of *Gnathostoma lamothei* in Tabasco, Mexico. Parasitol. Int..

[13] Wittner M., Turner J.W., Jacquette G., Ash L.R., Salgo M.P., Tanowitz H.B. (1989). Eustrongylidiasis—A parasitic infection acquired by eating sushi. N. Engl. J. Med..

[14] Narr L.L., O’Donnell J.G., Libster B., Alessi P., Abraham D. (1996). Eustrongylidiasis—A parasitic infection acquired by eating live minnows. J. Am. Osteopath. Assoc..

[15] Sirikulchayanonta V., Viriyavejakul P. (2001). Various morphologic features of *Gnathostoma spinigerum* in histologic sections: Report of 3 cases with reference to topographic study of the reference worm. Southeast Asian J. Trop. Med. Public Health.

[16] Tiwari S., Chayani N., Rautaraya B. (2009). Intraocular *Gnathostoma spinigerum*: A case report. Cases J..

[17] Bussaratid V., Dekumyoy P., Desakorn V., Jaroensuk N., Liebtawee B., Pakdee W., Wattanagoon Y. (2010). Predictive factors for *Gnathostoma* seropositivity in patients visiting the Gnathostomiasis Clinic at the Hospital for Tropical Diseases, Thailand during 2000–2005. Southeast Asian J. Trop. Med. Public Health.

[18] Kim J.H., Lim H., Hwang Y.S., Kim T.Y., Han E.M., Shin E.H., Chai J.Y. (2013). *Gnathostoma spinigerum* infection in the upper lip of a Korean woman: An autochthonous case in Korea. Korean J. Parasitol..

[19] Cui J., Wang Y., Wang Z.Q. (2013). Cutaneous gnathostomiasis with recurrent migratory nodule and persistent eosinophilia: A case report from China. Korean J. Parasitol..

[20] Eberhard M.L., Ruiz-Tiben E. (2014). Cutaneous emergence of *Eustrongylides* in two persons from South Sudan. Am. J. Trop. Med. Hyg..

[21] Chotmongkol V., Kitkhuandee A., Sawanyawisuth K. (2015). Spinal epidural hematoma and gnathostomiasis. Am. J. Trop. Med. Hyg..

[22] Diaz J.H. (2015). Gnathostomiasis: An Emerging Infection of Raw Fish Consumers in Gnathostoma Nematode-Endemic and Nonendemic Countries. J. Travel Med..

[23] Liu G.H., Sun M.M., Elsheikha H.M., Fu Y.T., Sugiyama H., Ando K., Sohn W.M., Zhu X.Q., Yao C. (2020). Human gnathostomiasis: A neglected food-borne zoonosis. Parasites Vectors.

[24] Mazzone A., Caffara M., Gustinelli A., Agnetti F., Sgariglia E., Lo Vaglio G., Quaglio F., Fioravanti M.L. (2019). Morphological and Molecular Characterization of Larval and Adult Stages of *Eustrongylides excisus* (Nematoda: Dioctophymatoidea) with Histopathological Observations. J. Parasitol..

[25] Menconi V., Riina M.V., Pastorino P., Mugetti D., Canola S., Pizzul E., Bona M.C., Dondo A., Acutis P.L., Prearo M. (2020). First Occurrence of *Eustrongylides spp*. (Nematoda: Dioctophymatidae) in a Subalpine Lake in Northwest Italy: New Data on Distribution and Host Range. Int. J. Environ. Res. Public Health.

[26] Novakov N., Bielic-Cabrilo O., Cirkovic M., Jubojevic D., Lujic J., Davidov I., Jovanovic M. (2013). Eustrongylidosis of European catfish (*Siluris glanis*). Bulg. J. Agric. Sci..

[27] Gasser R.B., Hoste H. (1995). Genetic markers for closely-related parasitic nematodes. Mol. Cell. Probes.

[28] Almeyda-Artigas R.J., Bargues M.D., Mas-Coma S. (2000). rDNA of *Gnathostoma* species (Nematoda): ITS-2 microsatellites and 5.8S gene secondary structure. Res. Rev. Parasitol..

[29] Ngarmamonpirat C., Waikagul J., Petmitr S., Dekumyoy P., Rojekittikhun W., Anantapruti M.T. (2005). Analysis of sequence variation in *Gnathostoma spinigerum* mitochondrial DNA by single-strand conformation polymorphism analysis and DNA sequence. Parasitol. Int..

[30] Xiong F., Wang G.T., Wu S.G., Nie P. (2009). Development of *Eustrongylides ignotus* (Nematoda: Dioctophmida) in domestic ducks (*Anas platyrhynchos domestica* (L.)). J. Parasitol..

[31] Nabavi R., Conneely B., McCarthy E., Good B., Shayan P., De-Waal T. (2014). Comparison of internal transcribed spacers and intergenic spacer regions of five common Iranian sheep bursate nematodes. Iran. J. Parasitol..

[32] Berland B. (1961). Nematodes from some Norwegian marine fishes. Sarsia.

[33] Diaz Camacho S.P., Willms K., Ramos M.Z., del Carmen de la Cruz Otero M., Nawa Y., Akahane H. (2002). Morphology of *Gnathostoma* spp. isolated from natural hosts in Sinaloa, Mexico. Parasitol. Res..

[34] Xiong F., Li W.X., Wu S.G., Zou H., Wang G.T. (2013). Molecular phylogeny and host specificity of the larval *Eustrongylides* (Nematoda: Dioctophmidae) from freshwater fish in China. J. Parasitol..

[35] Mascarenhas C.S., Müller G. (2015). Third-stage larvae of the enoplid nematode *Dioctophyme renale* (Goeze, 1782) in the freshwater turtle Trachemys dorbigni from southern Brazil. J. Helminthol..

[36] Zhu X.Q., Gasser R.B., Podolska M., Chilton N.B. (1998). Characterisation of anisakid nematodes with zoonotic potential by nuclear ribosomal DNA sequences. Int. J. Parasitol..

[37] Vogler A.P., Monaghan M.T. (2007). Recent advances in DNA taxonomy. J. Zool. Syst. Evol. Res..

[38] Marucci G., Interisano M., La Rosa G., Pozio E. (2013). Molecular identification of nematode larvae different from those of the *Trichinella* genus detected by muscle digestion. Vet. Parasitol..

[39] Sieu T.P., Dung T.T., Nga N.T., Hien T.V., Dalsgaard A., Waikagul J., Murrell K.D. (2009). Prevalence of *Gnathostoma spinigerum* infection in wild and cultured swamp eels in Vietnam. J. Parasitol..

[40] Sujata D.N., Renu B.S. (2013). Intraocular gnathostomiasis from coastal part of Maharashtra. Trop. Parasitol..

[41] Rojekittikhun W., Chaiyasith T., Nuamtanong S., Pubampen S., Maipanich W., Tungtrongchitr R. (2002). Gnathostoma infection in Nakhon Nayok and Prachin Buri, Central Thailand. Southeast Asian J. Trop Med. Public Health.

[42] Saksirisampant W., Thanomsub B.W. (2012). Positivity and intensity of *Gnathostoma spinigerum* infective larvae in farmed and wild-caught swamp eels in Thailand. Korean J. Parasitol..

[43] Jackson G.J., Bier J.W., Payne W.L., McClure F.D. (1981). Recovery of parasitic nematodes from fish by digestion or elution. Appl. Environ. Microbiol..

[44] Li D.M., Chen X.R., Zhou J.S., Xu Z.B., Nawa Y., Dekumyoy P. (2009). Short report: Case of gnathostomiasis in Beijing, China. Am. J. Trop. Med. Hyg..

[45] Hashimoto K., Watanobe T., Liu C.X., Init I., Blair D., Ohnishi S., Agatsuma T. (1997). Mitochondrial DNA and nuclear DNA indicate that the Japanese *Fasciola* species is *F. gigantica*. Parasitol. Res..

[46] Tamura K., Peterson D., Peterson N., Stecher G., Nei M., Kumar S. (2011). MEGA5: Molecular evolutionary genetics analysis using maximum likelihood, evolutionary distance, and maximum parsimony methods. Mol. Biol. Evol..

[47] Boonroumkaew P., Sanpool O., Rodpai R., Sadaow L., Somboonpatarakun C., Laymanivong S., Aung W.P.P., Un M., Laummaunwai P., Intapan P.M. (2019). Molecular identification and genetic diversity of *Gnathostoma spinigerum* larvae in freshwater fishes in southern Lao PDR, Cambodia, and Myanmar. Parasitol. Res..

[48] Eamsobhana P., Wanachiwanawin D., Roongruangchai K., Song S.L., Yong H.S. (2017). Genetic diversity of infective larvae of *Gnathostoma spinigerum* (Nematoda: Gnathostomatidae) in freshwater swamp eels from Thailand. J. Helminthol..

